# Intestinal Infarction and Portal Vein Thrombosis in a Patient with Henoch Schonlein Purpura

**DOI:** 10.1155/2012/672959

**Published:** 2012-10-14

**Authors:** Mekdess Abebe, Asha Patnaik, Frederick Miller, Heidi Roppelt, Nand K. Wadhwa, Mersema Abate, Edward P. Nord

**Affiliations:** ^1^Division of Nephrology, Department of Medicine, School of Medicine, State University of New York at Stony Brook, Stony Brook, NY 11794, USA; ^2^Division of Rheumatology, Department of Medicine, School of Medicine, State University of New York at Stony Brook, Stony Brook, NY 11794, USA; ^3^Department of Pathology, School of Medicine, State University of New York at Stony Brook, Stony Brook, NY 11794, USA

## Abstract

Henoch Schonlein purpura is a systemic vasculitis that commonly affects children and teenagers but also affects adults of all ages. In most instances it has a benign course. Organ involvement, particularly in adults, and notably the kidneys and gastrointestinal tract may require therapeutic intervention and may have a less favorable outcome. We report a case of a 58-year-old man who presented with purpura and who rapidly developed catastrophic intestinal vasculitis, leading to his demise.

## 1. Introduction 

Henoch Schonlein purpura (HSP) is a systemic vasculitis characterized by involvement of the skin, joints, kidney, and gastrointestinal tract [1–3]. It is primarily a disease of children but can occur at any age [2, 3]. In most cases, especially in children, it is a benign self-limiting disorder, but adults may require immunosuppressive therapy for complete recovery [2]. Gastrointestinal (GI) manifestations occur in 55–75% of adult HSP patients [1]. The most common GI symptoms include colicky abdominal pain, GI bleeding, and vomiting. Rarely, intussusception, bowel infarction and hemorrhagic ascites can complicate HSP [4–7]. Complete recovery with or without treatment usually occurs, and fatal complications are rare. We report an adult patient with HSP complicated by extensive infarction of the intestine, intraperitoneal hemorrhage, and portal vein thrombosis with a fatal outcome. 

## 2. Case Report 

 A 58-year-old white man was referred to the outpatient nephrology office by his gastroenterologist because of microscopic hematuria and proteinuria in the setting of an evolving purpuric rash. The rash had progressed proximally from both feet to his thighs, upper extremities and abdomen over the past two weeks. He complained of nausea, and vomiting for one day but denied hematemesis, melanotic stools, or arthralgia. His significant comorbidities included hypertension, untreated hepatitis C, and alcohol abuse. He took no medications.

On physical examination blood pressure was 105/62 mmHg, pulse was 84 beats/minute and regular, and temperature 37.7°C. The skin showed nontender, palpable purpuric lesions involving both upper and lower extremities and the abdominal wall. Lungs were clear to auscultation and the cardiovascular exam was unremarkable. On abdominal examination no organomegaly was appreciated. There was no pedal edema.

Laboratory tests revealed a white blood cell count of 13.1 × 10³/mcL, hemoglobin 14.7 g/dL, hematocrit 42.9%, and platelet count of 234 × 10³/mcL. Serum chemistry showed sodium 136 mEq/L, potassium 4.2 mEq/L, chloride 101 mEq/L, bicarbonate 24 mEq/L, blood urea nitrogen (BUN) 34 mg/dL and serum creatinine 2.0 mg/dL. Liver function tests revealed a total bilirubin of 1.3 mg/dL, direct bilirubin 0.5 mg/dL, ALT 75 units/L, AST 93 units/L, alkaline phosphatase 75 units/L, total protein 7.6 g/dL and albumin 3.7 g/dL. Hepatitis B core antibody, hepatitis B surface antibody and hepatitis C antibody were all positive. Urinalysis demonstrated trace hematuria and proteinuria. He was sent to the emergency room for further evaluation.

**Figure 1:**
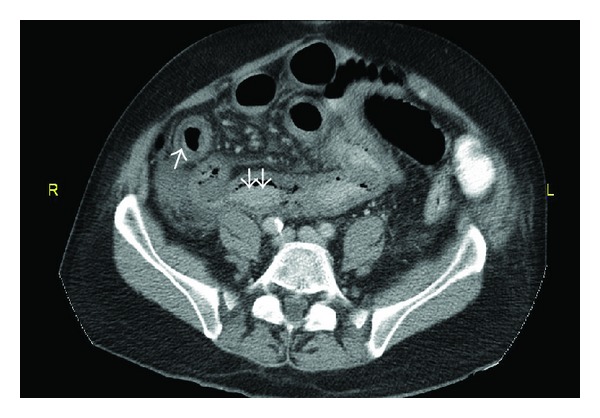
Computed tomography (CT) of the abdomen and pelvis with IV contrast showing marked thickening of the terminal ileum.

On admission the patient was started on intravenous fluids and additional tests were obtained. Antinuclear antibody (ANA) and serum complements (C_3_ and C_4_) were normal and antineutrophil cytoplasmic antibody (ANCA) and cryoglobulins were negative. The serum creatinine improved to 0.75 mg/dL on hydration alone. Two days later he complained of diffuse abdominal pain and dark stool followed by hematochezia. He remained afebrile with a blood pressure of 120/50 mmHg. On physical examination he had significant right upper abdominal quadrant and epigastric tenderness. Esophagogastroduodenoscopy (EGD) was performed that failed to reveal a lesion that could account for his symptoms. A biopsy of the stomach mucosa demonstrated mild chronic inflammation. Computerized tomography (CT) scan of the abdomen was next performed and showed marked thickening of the terminal ileum (Figure 1). Abdominal pain intensified, and the patient became tachycardic and tachypneic with a leukocytosis of 23 × 10³/mcL, with deteriorating renal function (BUN 54 mg/dL, serum creatinine of 2.0 mg/dL) and evolving acidosis (bicarbonate 17 mEq/L). An emergency exploratory laparotomy was performed which revealed gangrenous bowel from the beginning of the ileum to the transverse colon. Small bowel resection (90 cm of ileum) and right hemicolectomy (including appendix which was involved) with end jejunostomy were performed.

**Figure 2:**
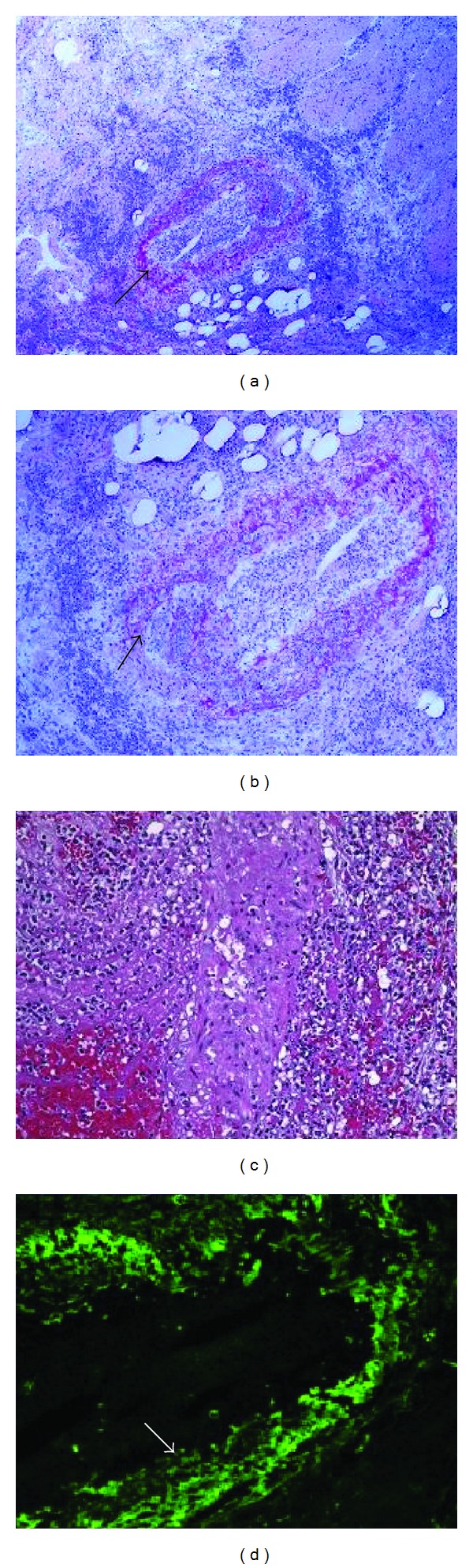
(a) A small artery in the intestinal submucosa showing necrotizing arteritis (H&E). (b) A higher power view of the same vessel showing early fibrin formation in the lumen (arrow). (c) A high power view of another vessel emphasizing the necrosis and the largely neutrophil reaction (H&E). (d) A randomly selected artery illustrating the heavy deposition of IgA (immunofluorescence with an alpha chain-specific antibody).

Histopathologic examination of the resected intestinal tissue demonstrated an extensive necrotizing vasculitis with IgA deposition characteristic of HSP (Figure 2). Of particular note was the very widespread (perhaps 50%) involvement of medium sized vessels, particularly arteries, with extensive necrosis and secondary ischemic injury to the intestines. Small arteries, arterioles, and venules were also involved. Heavy granular IgA deposition was seen with no staining for IgG or IgM. Lesser amounts of C_3_ paralleled the IgA deposition.

Intravenous methylprednisolone 1 g daily for three days was administered and then 30 mg every 12 hours. Postoperatively he continued to have an elevated white blood cell count and persistent fever, but his renal function improved. A repeat CT scan of the abdomen revealed persistent small bowel wall thickening. A second exploratory laparotomy was performed, but no additional ischemic bowel was identified. The patient was continued on broad-spectrum antibiotics and maintained on a ventilator due to respiratory failure. Eight days after the second laparotomy he developed severe lactic acidosis, right upper abdominal quadrant tenderness jaundice and worsening renal function. Ultrasound of the right upper quadrant showed findings compatible with portal vein thrombosis and cholelithiasis. Repeat CT scan of the abdomen showed increased ascites with intraperitoneal hemorrhage. Hemodialysis was initiated due to worsening oliguric acute kidney injury. His condition continued to deteriorate, and he died one day later, a total of 27 days after admission.

## 3. Discussion

HSP is a systemic small-vessel vasculitis that is mainly a disease of early childhood [1–3, 10]. Overall prognosis is good in both children and adults, with one study showing complete recovery occurring in 94% of children and 89% of adults [2]. In this regard, recovery is usually spontaneous in children, whereas in adults immunosuppressive therapy may be required in up to 63% of cases [2]. We present a case of a middle-aged man who developed extensive gangrenous bowel and intraperitoneal hemorrhage due to HSP vasculitis, with a fatal outcome.

GI symptoms are one of the commonest manifestations of HSP in adults, involving 55–75% of individuals [1]. In a retrospective analysis of 115 adults with the diagnosis of HSP, GI symptoms were reported in 90 patients (78.2%), abdominal pain being the most common (89%) followed by vomiting and GI bleeding [4]. Furthermore, 24% of patients had GI symptoms prior to the development of a cutaneous rash [4]. In another retrospective study involving 116 children and 46 adults, adults had a lower frequency of GI involvement (5%) at disease onset, but during the clinical course GI involvement was the same in both age groups (56.5% of adults versus 63.8% of children) [2]. In a retrospective analysis of 250 adults with HSP, GI involvement was observed in 48%. In 13 of these cases (11%) serious GI complication developed requiring transfusion or surgery or leading to death [3]. The small intestine is the most common site involved, terminal ileum (60%) and the second portion of the duodenum (53%). The rectum (80%) is the most frequently affected areas in the lower GI tract [4, 8]. Endoscopic findings include mucosal congestion, redness, petechiae, multiple ulcers, and hemorrhagic erosions [4, 8, 9]. Rarely, severe GI complication such as bowel infarction, perforation, fistula, intussusceptions (ileoileal), hemorrhagic ascites, and pancreatitis can occur [4–7, 10]. 

A number of inciting antigens have been implicated in the causation of HSP. These include bacterial and viral infections, vaccinations, drugs, malignancy, and autoimmune phenomena. The pathogenic mechanism of organ involvement in HSP is thought to be due to the deposition of antigen antibody complexes in the small vessel walls. This leads to activation of the alternate complement pathway leading to neutrophil accumulation resulting in inflammation and vasculitis [9, 10]. IgA is the antibody class most often seen in the immune complex [10]. The presence of hepatitis C virus could have been a triggering factor in our patient as has been suggested by others [10]. 

To the best of our knowledge, the extensive small and large intestine infarction with gangrene that led to a fatal outcome in our patient is rarely seen in HSP. In this case, in contrast to the majority of those reported, the arteritis was extensive and involved medium sized vessels (0.3–0.5 cm) in many areas as well as the small arteries, arterioles, and venules. The involvement of the transverse colon and appendix as seen in our patient is also rare in HSP. An additional complicating factor in this case was the occurrence of portal vein thrombosis. In reviewing the literature, there is only one reported case of portal vein thrombosis complicating HSP [11]. However, in the patient presented here, we are unable to conclude whether the portal vein thrombosis was associated with underlying hepatitis C or was a complication of HSP. Hepatitis C, without cirrhosis would be unlikely to cause portal vein thrombosis.

The mainstay of therapy in HSP with severe organ involvement has been high-dose steroids. Other immunosuppressive agents such as cyclophosphamide, azathioprine, and mycophenolate mofetil have also been used [12, 13]. There has been only one prospective randomized trial comparing steroid therapy with and without cyclophosphamide in cases of severe visceral HSP [14]. The results of that study showed that addition of cyclophosphamide to steroids did not improve the outcome of the disease as compared to steroids alone. Our patient was treated with pulse steroids alone, with no significant improvement.

In conclusion, given the potential severity and fatal outcome of HSP involving the intestines in adults, prompt and early recognition of this entity is crucial. The mainstay of therapy remains high-dose steroids, with little evidence to support the use of other immunosuppressive agents. In catastrophic circumstances as described in this instance, even high-dose steroids may not alter the course of events.

## References

[1] Saulsbury FT (2007). Clinical update: Henoch-Schönlein purpura. *Lancet*.

[2] Blanco R, Martínez-Taboada VM, Rodríguez-Valverde V, García-Fuentes M, González-Gay MA (1997). Henoch-Schonlein purpura in adulthood and childhood: two different expressions of the same syndrome. *Arthritis and Rheumatism*.

[3] Pillebout E, Thervet E, Hill G, Alberti C, Vanhille P, Nochy D (2002). Henoch-Schönlein Purpura in adults: outcome and prognostic factors. *Journal of the American Society of Nephrology*.

[4] Zhang Y, Huang X (2008). Gastrointestinal involvement in Henoch-Schönlein purpura. *Scandinavian Journal of Gastroenterology*.

[5] Akbar DH (2000). Fatal complication of Henoch-Schonlein purpura: case report and literature review. *Saudi Journal of Gastroenterology*.

[6] Ebert EC (2008). Gastrointestinal manifestations of Henoch-Schonlein purpura. *Digestive Diseases and Sciences*.

[7] Carmichael P, Brun E, Jayawardene S, Abdulkadir A, O’Donnell PJ (2002). A fatal case of bowel and cardiac involvement in Henoch-Schönlein purpura. *Nephrology Dialysis Transplantation*.

[8] Hamzaoui A, Melki W, Harzallah O, Njim L, Klii R, Mahjoub S (2011). Gastrointestinal involvement revealing Henoch Schonlein purpura in adults: report of three cases and review of the literature. *International Archives of Medicine*.

[9] Kato S, Ebina K, Naganuma H, Sato SI, Maisawa SI, Nakagawa H (1996). Intestinal IgA deposition in Henoch-Schönlein purpura with severe gastro-intestinal manifestations. *European Journal of Pediatrics*.

[10] Sohagia AB, Gunturu SG, Tong TR, Hertan HI (2010). Henoch-schonlein purpura—a case report and review of the literature. *Gastroenterology Research and Practice*.

[11] Choi SJ, Park SK, Uhm WS (2002). A case of refractory Henoch-Schönlein purpura treated with thalidomide. *The Korean Journal of Internal Medicine*.

[12] Kellerman PS (2006). Henoch-Schönlein purpura in adults. *American Journal of Kidney Diseases*.

[13] Nikibakhsh AA, Mahmoodzadeh H, Karamyyar M (2010). Treatment of complicated henoch-schnlein purpura with mycophenolate mofetil: a retrospective case series report. *International Journal of Rheumatology*.

[14] Pillebout E, Alberti C, Guillevin L, Ouslimani A, Thervet E (2010). Addition of cyclophosphamide to steroids provides no benefit compared with steroids alone in treating adult patients with severe Henoch Schönlein Purpura. *Kidney International*.

